# Hydroxychloroquine induces endothelium-dependent and endothelium-independent relaxation of rat aorta

**DOI:** 10.55730/1300-0144.5382

**Published:** 2022-01-22

**Authors:** Seyfullah Oktay ARSLAN, Muhammed Fatih DOĞAN, Saliha Ayşenur ÇAM, Ibraheem Akram OMAR, Fatma UYSAL, Ali PARLAR, Ahmet Cenk ANDAÇ, Oğuzhan YILDIZ

**Affiliations:** 1 Department of Medical Pharmacology, Faculty of Medicine, Ankara Yıldırım Beyazıt University, Ankara, Turkey; 2 Department of Medical Pharmacology, Faculty of Medicine, Pamukkale University, Denizli, Turkey; 3 Department of Medical Pharmacology, Faculty of Medicine, Adıyaman University, Adıyaman, Turkey; 4 Department of Medical Pharmacology, Faculty of Medicine, Yeditepe University, İstanbul, Turkey; 5 Department of Medical Pharmacology, Gülhane Faculty of Medicine, Health Sciences University, Ankara, Turkey

**Keywords:** Hydroxychloroquine, nitric oxide, calcium channels, vasodilation

## Abstract

**Background/aim:**

Hydroxychloroquine (HCQ) is an antimalarial that is widely used in the management of rheumatoid arthritis and other autoimmune diseases. In this study, we aimed to examine the vascular effects of HCQ on rat aorta (RA).

**Materials and methods:**

The RA rings were suspended in isolated organ baths and tension was recorded isometrically. HCQ-induced relaxations were tested in the presence of the nitric oxide synthase inhibitor, nitro-L-arginine methyl ester (L-NAME, 100 mM); the cyclooxygenase enzyme inhibitor, indomethacin (10 mM); the calcium (Ca^2+^) ion channel blocker, nilvadipine (10 μM); and the K^+^ ion channel inhibitors, tetraethylammonium (1 mM), glibenclamide (10 mM), 4-aminopyridine (1 mM), and barium chloride (30 mM). The effect of HCQ on Ca^2+^ channels was examined using Ca^2+^-free Krebs solution, and adding calcium chloride (CaCl_2_, 10^−5^–10^−2^ M) cumulatively to baths incubated with HCQ.

**Results:**

Removing the endothelium resulted in less relaxation of RA rings compared to endothelium-intact rings (p < 0.05). The effect of endothelium was supported by using L-NAME where HCQ produced-vasorelaxation was decreased (p < 0.05). The contraction of vascular rings was inhibited to a significant degree following the addition of CaCl_2_, PE, or KCl on HCQ-incubated RA rings (p < 0.05). The incubation of the RA rings with the Ca^2+^ channel blocker, the K^+^ channel blockers, and the COX inhibitor, indomethacin did not significantly affect vascular relaxation induced by HCQ.

**Conclusion:**

HCQ produced relaxation of RA rings. The relaxation mechanism differs according to the concentration of HCQ. At concentrations of 10^−6^ and 10^−5^ M, the relaxation is endothelium-dependent and mediated by NO. We strongly suggest that Ca^2+^ channel inhibition is involved at concentrations of 10^−5^ and 10^−4^ M, as well as NO.

## 1. Introduction

Hydroxychloroquine (HCQ), a 4-aminoquinoline-type antimalarial drug, was first introduced into the market in 1955 several years after chloroquine (CQ), another 4-aminoquinoline-type antimalarial drug that differs from HCQ by a missing hydroxyl group at the diethylamino terminal, was taken into the clinical practice in 1947 [1]. Both HCQ and CQ had been alternatively developed as safer antimalarial drugs in attempts to replace quinaqrine which has a lower therapeutic index when applied in the treatment of malaria. Discovery of the ameliorative effects of quinacrine on cutaneous lupus and inflammatory arthritis has initiated intensive research on HCQ and CQ whose antiinflammatory results were published in the early 1950s [2] leading to their medical applications in the treatment of rheumatoid arthritis, systemic lupus erythematosus (SLE), and other autoimmune diseases [3]. Besides its antiinflammatory activity, HCQ has been shown to take effect on various body systems [4].

Some cardiac adverse effects, mainly QT interval prolongation and cardiomyopathy, are observed in patients receiving HCQ. Some researchers have discussed beneficial effects of HCQ on the cardiovascular profile of rheumatoid arthritis patients [5,6]. Bengtsson et al. investigated microvascular responses in the skin in SLE patients taking antimalarial drugs. Arthritis patients taking HCQ responded more strongly to the endothelium-independent vasodilating effect of sodium nitroprusside as compared to those not taking HCQ [7].

The endothelium layer of vasculature is primarily involved in the regulation of vascular tone via the syntheses and release of endothelium-derived relaxing factors such as nitric oxide (NO) and endothelium-derived contracting factors [8]. Abiose et al., who studied the vasodilation effect of CQ on dorsal hand veins, suggested that the venodilation effect of CQ is mediated by NO and histamine release [9]. Adegunluye et al. partly related the vasodilating effect of mefloquine on rat aortic rings to the endothelium, which could have mediated the inhibition of calcium influx from extracellular medium as suggested by the authors [10]. Aziba et al. studied the effects of CQ on rat thoracic aorta and suggested that the vasorelaxation caused by CQ was neither mediated by hyperpolarization nor by endothelium-derived relaxing factors nor via the cGMP pathway. As a result, the authors reached a conclusion that CQ may exert its vasodilating effect via regulatory proteins involved in the modulation of the contractile system [11]. Potassium (K^+^) ion channel activity is another important regulator of vascular tone. Five different types of K+ channels are expressed in the vasculature: big conductance Ca^+2^-activated (BK_Ca_), voltage-activate (K_V_), ATP-sensitive (K_ATP_), inward rectifier (Kir), and two-pore domain (K_2P_) K^+^ channels. These channels undergo changes in expression and/or activity during major vascular diseases such as hypertension and atherosclerosis [12].

Gomez et al. reported that chronic hydroxychloroquine treatment has decreased hypertension and improved endothelial dysfunction in NZBWF1 lupus mice [13]. In this study, we aimed to address the mechanism of the effects of HCQ on rat aorta via conducting competitive binding studies on K^+^ and Ca^+2^ ion channels as well as the enzymes COX and eNOS involved in regulating the vascular tone.

## 2. Materials and methods

### 2.1. Animals

Female Wistar-Albino rats (16–20 weeks old, weighing 200–250 g) were housed under controlled conditions (12 h light, 12 h dark, temperature: 20–22 °C; relative air humidity: 50 ± 5%) in laboratory animal production and research center Adıyaman university, Adıyaman, Turkey. Diet and drinking water were given ad libitum.

All animal procedures were performed with the approval of the Adıyaman University Animal Care and User Committee (approval number: 19, accredited on June 25th, 2020) in accordance with the User Guidelines for the Laboratory Animal Production and Research Center at Adıyaman University.

### 2.2. Preparation of aortic rings

Rats were anesthetized by intraperitoneal injection of xylazine (10 mg/kg) and ketamine (90 mg/kg). The chest of each rat was opened to reveal the aorta. The aorta was carefully cut out and placed into Krebs-Henseleit solution [containing NaCl (118 mM), NaHCO_3_ (25 mM), D-Glucose (10 mM), KCl (4.7 mM), MgSO_4_.7H_2_O (1.2 mM), K_2_HPO_4_ (1.2 mM), and CaCl_2_ (2.5 mM)], from which fat and connective tissues were cleaned off fat and connective tissue. The aorta was then sliced into 3–5 mm long ring segments. Rat aortic (RA) rings were mounted on wire hooks in a 10-mL organ bath (COMMAT LTD, Turkey) containing Krebs-Henseleit solution and continuously bubbled with a mixture of 95% O_2_ (g) and 5% CO_2_ (g). The organ bath was kept at 37 °C. A transducer system equipped with the Biopac Student Lab 4.0 (Biopac Systems, Inc., USA) program was used to record the contraction/relaxation responses of the tissue rings to the chemicals.

### 2.3. Setting basal tension

At the beginning of each experiment, 1.5 g of passive resting tension was applied on the ring segments which were allowed to equilibrate for 90 min. The Krebs-Henseleit solution was replenished every 15 min during the equilibrium period.

### 2.4. Testing viability of the vessels

After tension equilibration, the viability of the aortic ring segments was tested by adding potassium chloride (KCl) at a final concentration of 45 mM into the organ bath. Vessels that contracted by at least 1 g of force were used in the experiments.

### 2.5. Effects of HCQ on intact and denuded endothelium

To determine a possible role of endothelium in HCQ-mediated vascular relaxation, aortas taken from rats were sliced into two 3–5 mm long ring segments. The endothelial layer of one of the aortic ring segments was mechanically disrupted by rubbing the vessel gently against a stainless-steel wire. The vascular ring segments were then treated with 1 μM of aqueous phenylephrine (PE) solution in the organ bath. Once vessel contractions reached a plateau phase, acetylcholine (ACh) was added to the organ bath at a final concentration of 10^−5^ M. Aortic ring segments that showed at least 30% relaxation were considered intact endothelium. While those that failed to relax more than 10% were accepted as denuded endothelium. The aortic ring segments were rinsed with Krebs-Henseleit solution three times and left in the organ bath to rest for 30 min. Then, the resting aortic ring segments were recontracted by addition of PE (1 μM) into the organ bath, to allow them to reach a plateau phase, followed by addition of the increasing final concentrations of 10^−6^ M, 10^−5^ M, 10^−4^ M, and 10^−3^ M HCQ, respectively. Vascular responses were recorded at each increment of HCQ concentration.

### 2.6. Effects of HCQ on KCl and PE contractions

In this part of the study, the inhibition of the contractive effect of KCl and PE on the RA ring segments of HCQ at different concentrations was analyzed. After the equilibration period, different RA rings were incubated with HCQ 10^−6^ M, 10^−5^ M, 10^−4^ M, or 10^−3^ M (one concentration in each organ bath) for 20 min, then KCl 45 mM or PE 1 μM were added. The contractions were recorded and compared with HCQ-free control group.

### 2.7. Effect of potassium or calcium channel inhibitors on aortic vessel response to HCQ

Possible roles of K^+^ or Ca^2+^ ion channels in HCQ-mediated vasorelaxation were determined using the RA ring segments that equilibrated and passed the viability test. The selected RA ring segments were separately incubated for 20 min in a 1 mM solution of tetraethylammonium (TEA), a BK_Ca_ and K_V_ inhibitor, a 1 mM solution of 4-aminopyridine (4-AP), a K_V_ inhibitor, a 30 μM solution of barium chloride (BaCl_2_), an inward rectifier K^+^ channel (K_IR_) inhibitor, a 10 μM solution of glibenclamide (GLY), an ATP-sensitive K^+^ channel inhibitor and a 10 μM solution of nilvadipine, a Ca^2+^ channel inhibitor. The RA ring segments were then contracted in 1 μM solutions of PE to reach a steady contraction phase. This followed incremental additions of HCQ from the stock solution into the organ baths to yield 10^−6^, 10^−5^, 10^−4^, and 10^−3^ Molar concentrations of HCQ, allowing 20 min time intervals in between the HCQ concentration increments for recording vasorelaxation.

### 2.8. Role of endothelium in vasodilatory effect of HCQ

Here, effects of nitric oxide synthase (NOS) and cyclooxygenase (COX) inhibitors on aortic responses to HCQ were tested. RA ring segments that equilibrated and passed the viability test were incubated separately. RA rings were incubated with a 10 μM solution of indomethacin (INDO), a cyclooxygenase (COX) inhibitor, or a 100 mM solution of *N(*ω)-nitro-L-arginine methyl ester (L-NAME), a nitric oxide synthase (NOS) inhibitor, for 30 minutes. As a control, one RA ring segment was processed without NOS or COX inhibitors. To contract the vessels, a certain amount of a stock solution of PE was then added to give rise to 1 μM PE concentration in the organ baths. After the contractions reached a plateau phase, certain amounts of HCQ (from a stock) solution were incrementally added to the organ baths at certain 10^−6^ M, 10^−5^ M, 10^−4^ M, and 10^−3^M, respectively, in the organ baths. Vascular tensions were measured in between the time intervals of HCQ addition (5 min after each addition of HCQ) and compared to the control as percentage of maximum contraction.

### 2.9. Effect of HCQ on CaCl_2_ induced contractions

In order to determine the effect of HCQ on Ca^2+^ influx into smooth muscle/endothelial cells via L-type calcium channels, CaCl_2_ concentration response curves (from 10 μM to 10 mM) versus aortic vessel tension were plotted in absence and presence of HCQ as described previously [14]. RA ring segments that equilibrated and passed the viability test were washed out with Ca^+2^-free Krebs solution for 3 times at 10-min intervals, followed by incubation in 45 mM KCl for 20 min in absence or presence of HCQ at varying concentrations (10^−6^M, 10^−5^ M, 10^−4^ M, and 10^−3^ M). Finally, cumulative concentration-response curves were constructed by adding CaCl_2_ (10 μM to 10 mM) to organ baths.

### 2.10. Chemicals

HCQ was isolated and purified by ultrasonic extraction out of HCQ tablets (Plaquenil^®^ by SANOFI, Paris, France), using CH_3_OH:H2O (50:50) solvent system. The extraction solvent system was finally evaporated under vacuum by a rotary evaporator to yield pure HCQ as off-white powder. GLY, 4-AP, and TEA were purchased from Sigma Chemical Co. (St. Louis, MO, USA). KCl was obtained from Carlo Erba reagents S.A.S (France). Dimethyl sulfoxide was purchased from ISOLAB (Wertheim, Germany). Indomethacin was purchased from Deva (Istanbul, Turkey). L-NAME was obtained from Cayman chemical (Ann Arbor, MI, USA). BaCl_2_ was obtained from ZAG chemicals (Istanbul, Turkey).

### 2.11. Statistical methods

Relaxation responses of RA ring segments to HCQ and ratios of KCl-, PE-, and CaCl_2_-induced contractions were expressed in percent values. SPSS 18.0 (SPSS Inc., Chicago, IL, USA) was used for statistical analysis. GraphPad Prism 7.04 (GraphPad Software, San Diego, CA, USA) was used for graphical presentations. All results are expressed in mean ± standard error of the mean (SEM). The sample size calculation was based on a preliminary study. The appropriate sample size was found to be 6 using online software (Experimental Design Assistant^1^; alpha=0.05, power=85%). A standard effect size of 1 (medium effect) was chosen based on Cohen’s d guidelines. Standard deviation (SD) value was found to be 0.6. It was calculated based on data from the preliminary study using SPSS 18.0. A one-sided test was performed. Data normality test with Shapiro–Wilk found a value more than 0.05 so that the data is said to be normally distributed. Furthermore, the homogeneity test obtained a value more than 0.05; accordingly, the research sample was confirmed to be homogenous.

Statistical significance between 2 independent groups was determined by independent sample t test. One-way analysis of variance test (ANOVA) and Scheffe’s post hoc test were used for comparing multiple groups. A probability level of p < 0.05 was considered statistically significant.

## 3. Results

### 3.1. Response of intact and denuded endothelium to HCQ

Initially, it is essential to address whether, contracted or relaxed, the endothelial cells are involved in a response given to HCQ administration.

**Figure 1:**
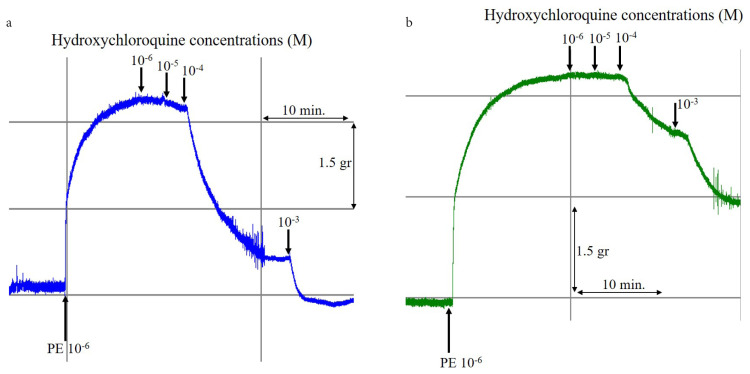
Continuous transducer recording for HCQ (10^−6^ M, 10^−5^ M, 10^−4^ M, and 10^−3^M)-induced relaxations of (A) intact and (B) denuded endothelium of RA ring segments, which were precontracted via PE (10^−6^ M).

**Figure 2:**
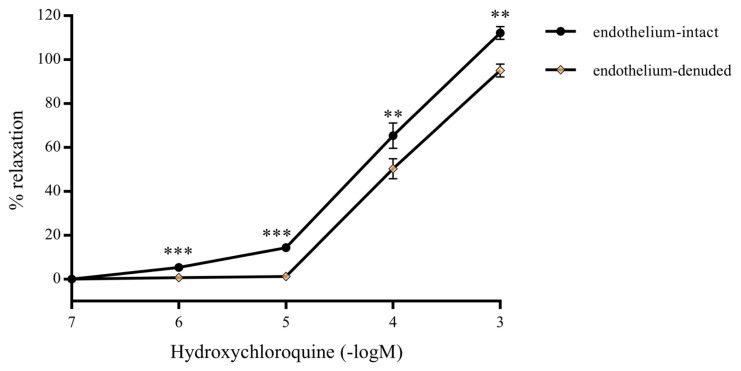
Plots of percent vascular relaxation versus hydroxychloroquine concentrations (10^−6^ M, 10^−5^ M, 10^−4^ M, 10^−3^ M) applied on denuded and intact endothelium of RA ring segments previously precontracted with 10^−6^ M of phenylephrine solution in the organ bath. Values are expressed in mean ± SEM (n = 7). *** and ** denotes p < 0.001 and ** p < 0.01, respectively.

The response of intact and denuded endothelium exposed to HCQ was determined in presence of lower (10^−6^ to10^−5^ M) and higher (10^−4^ to 10^−3^ M) concentrations of HCQ. It was determined that all concentrations of HCQ were able to relax the intact endothelium of the precontracted RA ring segments (Figure 1A), while the endothelium-denuded precontracted RA ring segments exhibited very little or virtually no relaxation (p < 0.001) at lower HCQ concentrations (Figure 1B). Relaxation of the denuded endothelium became more prominent, and yet less than that of the intact endothelium, when higher HCQ concentrations were used (Figure 2). HCQ was not observed to influence resting RA ring segments. The results were statistically significant (p < 0.01).

### 3.2. Effect of HCQ on vascular contractions induced by PE, KCl, and CaCl_2_

Having found that HCQ induces aortic vasodilation, the effect of HCQ was further tested on RA ring segments precontracted by PE, an α1-adrenergic receptor agonist, KCl, and CaCl_2_.

**Figure 3:**
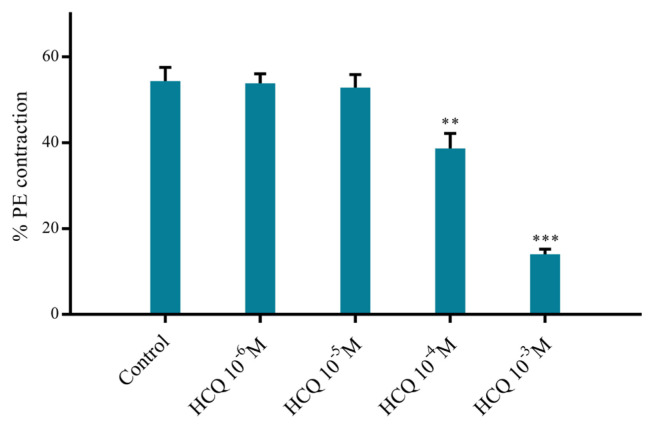
Percent phenylephrine (PE, 10^−3^ M)-induced contraction of RA ring segments incubated at increasing concentrations of hydroxychloroquine (HCQ, 10^−6^–10^−3^ M). Percent contraction values are given in mean ± SEM (n = 6). *** denotes p < 0.001 and ** indicates p < 0.01 as compared to the control group.

**Figure 4:**
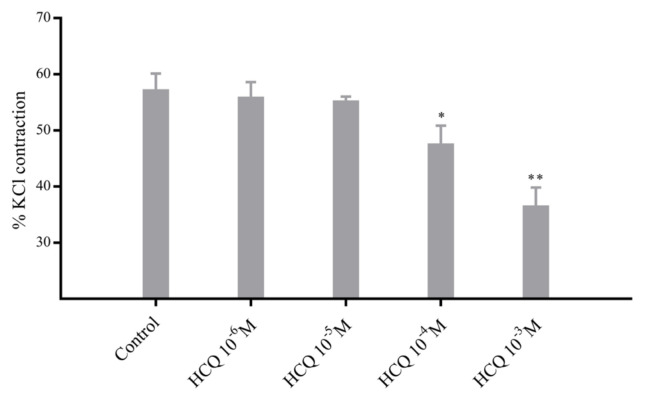
Percent potassium chloride (KCl, 10^−3^ M)-induced contraction of RA ring segments incubated at increasing concentrations of hydroxychloroquine (HCQ, 10^−6^–10^−3^ M). Percent contraction values are given in mean ± SEM (n = 6). ** denotes p < 0.01 and ** indicates p < 0.05 as compared to the control group.

Addition of suitable amounts of PE or KCl from their stock solutions to yield final concentrations of 10^−6^ M or 45 mM, respectively, in organ baths resulted in significantly less contractions (see Figures 3 and 4) when RA ring segments were incubated in 10^−4^ M HCQ, with p < 0.01 and p < 0.05 values for the PE (Figure 3) and KCl (Figure 4) groups, respectively, as determined in reference to the control and 10^−3^ M HCQ, with p < 0.001 and p < 0.01 values for the PE (Figure 3) and KCl (Figure 4) groups, respectively, as determined in reference to the control, where the control group included no HCQ.

**Figure 5:**
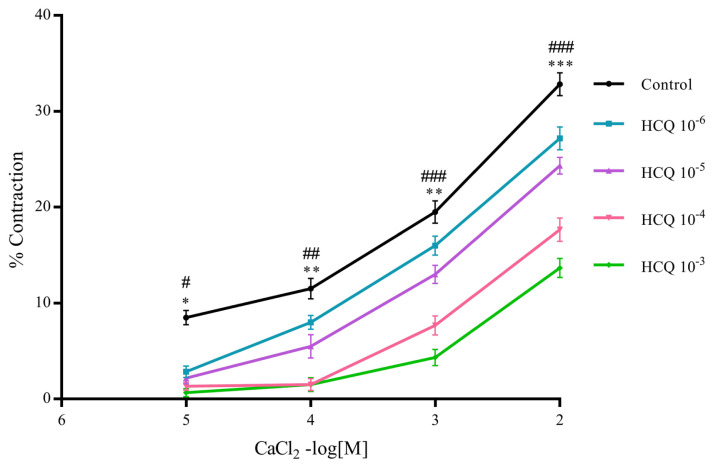
Percent calcium chloride (CaCl_2_, 10^−5^–10^−2^ M)-induced contraction of RA ring segments incubated at increasing concentrations of hydroxychloroquine (HCQ, 10^−6^–10^−3^ M). Percent contraction values are given in mean ± SEM (n = 6). * is p < 0.05 at 10^−4^ M CaCl_2_, ** is p < 0.01 at 10^−4^–10^−3^ M CaCl_2_, and *** is p < 0.001 at 10^−2^ M CaCl_2_ as compared to the control group. ^#^ p < 0.05, ^##^ p < 0.01, and ^###^p < 0.001 for HCQ 10^−3^ M vs. control group.

Figure 5 exhibits plots of percent CaCl_2_ induced contractions of RA ring segments versus increasing concentrations of CaCl_2_ (10^−5^–10^−2^ M), all incubated separately in different concentrations of HCQ solution (10^−6^–10^−3^ M). As far as Ca^2+^-induced aortic contractions are concerned, the relaxation property of HCQ takes more prominent effect at relatively higher concentrations of HCQ, specifically at 10^−4^ M (with p < 0.05, p < 0.01, p < 0.01, and p < 0.001 values, respectively, for the RA ring segments pre-contracted in 10^−5^ M, 10^−4^ M, 10^−3^ M, and 10^−2^ M Ca^2+^ solutions respectively) and 10^−3^ M (with p < 0.05, p < 0.01, p < 0.001, and p < 0.001 values, respectively, for the RA ring segments precontracted in 10^−5^ M, 10^−4^ M, 10^−3^ M, and 10^−2^ M Ca^2+^ solutions, respectively) (Figure 5), as compared to the control group in which no HCQ was used.

### 3.3. Effects of inhibitors of NOS, COX, calcium channels, and potassium channels on HCQ-induced vascular relaxation

**Figure 6:**
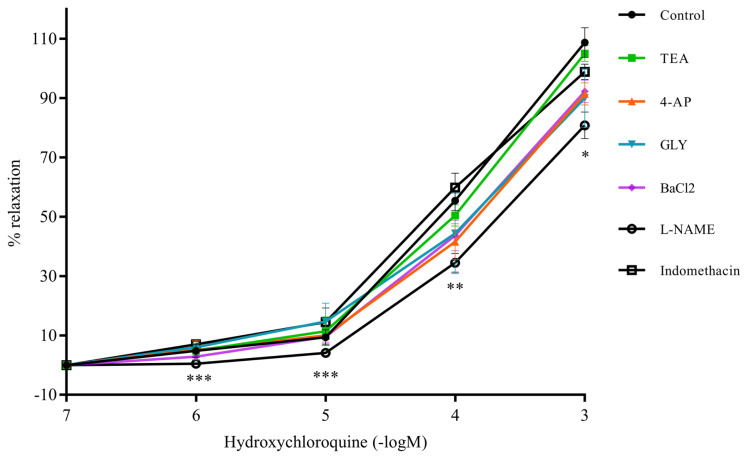
Relaxation of RA rings in response to hydroxychloroquine (10^−6^–10^−3^M) after incubation with K^+^ channel blockers: TEA, 4-AP, GLY, BaCl_2_, the COX enzyme inhibitor indomethacin or the endothelial NOS inhibitor L-NAME. Values are expressed as mean ± SEM (n = 7). ***p < 0.001 and *p < 0.05 vs. control group.

**Figure 7:**
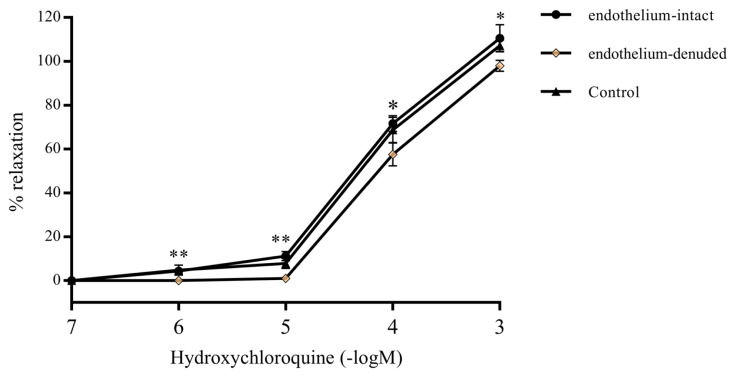
Relaxation of RA rings in response to hydroxychloroquine (10^−6^ M–10^−3^M) after incubation with Ca2+ channel blocker, nilvadipine (10 μM). Values are expressed as mean ± SEM (n = 6). **p < 0.01 and *p < 0.05 for endothelium-denuded group vs. control group.

Whether K^+^ channels, Ca^2+^ channels, COX, or NOS have any role in the mechanism of HCQ-induced relaxation was investigated by using their inhibitors. Blocking the four K^+^ channels, namely: BK_Ca_, K_V_, K_ATP_ and K_IR_, has no effect on the relaxation obtained with HCQ. Similarly, incubating RA rings with the COX inhibitor indomethacin has failed to show statistically significant differences in HCQ-induced relaxation. However, as shown in Figure 6, the inhibition of NOS by incubating RA rings with L-NAME resulted in less vascular relaxation in response to HCQ, which was statistically significant compared to the control group at HCQ concentrations; 10^−6^ M (p < 0.001), 10^−5^ M (p < 0.001), 10^−4^ M (p < 0.01), and 10^−3^ (p < 0.05). Nilvadipine incubation relaxed both endothelium-intact and -denuded rings; and the relaxation of -denuded rings was lower compared to the control group (p < 0.01 for 10^−6^ and 10^−5^ M, and p < 0.05 for 10^−4^ and 10^−3^ M) (Figure 7).

## 4. Discussion

Chloroquine and hydroxychloroquine are antimalarial drugs that are also commonly prescribed for rheumatoid arthritis, SLE, and other autoimmune diseases. Regarding their effects on vascular smooth muscle, CQ had taken the bigger share of research, whereas HCQ was not investigated that much; possibly because the former was more widely used in the clinical setting. Capel et al. studied the in vitro effect of cumulative doses of HCQ (1–10 μM) on spontaneous beating rate in mouse atrial preparations. However, the vascular effects of HCQ have not been studied [15]. In this study, we found that HCQ relaxes RA rings precontracted with phenylephrine in a concentration-dependent manner. To find out the mechanism underlying this relaxing effect, we made a series of experiments on endothelium-denuded and endothelium-intact rings, and used blockers of K^+^ channels, Ca^2+^ channels, and inhibitors of COX and eNOS enzymes. Our laboratory results indicate that HCQ induces vasodilation partly via endothelium and partly due to endothelium-independent mechanisms.

The endothelium layer of blood vessels is an extremely important element in vascular dilation used both by endogenous substances, e.g., acetylcholine, substance P, ATP, bradykinin [16], and exogenous compounds [17,18]. Our results show a considerable role of the endothelium in mediating HCQ-induced relaxation, exhibited by less relaxation of the RA rings in response to HCQ at the tested concentrations, 10^−6^ M–10^−3^ M, when the endothelium is removed. Adegunloye et al. studied the effects of mefloquine—another congener of HCQ—on rat aortic rings and suggested that mefloquine-induced relaxation is partly endothelium-dependent [10]. Aziba et al., who studied the effect of chloroquine on RA rings, stated that vascular relaxation obtained by chloroquine was endothelium-independent. Nevertheless, they denoted that uncovering detailed mechanism of relaxation needs more research [11]. Since the endothelium contributes to vascular relaxation through many factors, we went forward to determine which one is the main player. While relaxation of RA rings by HCQ was not affected by incubation with the COX inhibitor, indomethacin, our results show appreciable reduction in HCQ-induced relaxation in RA rings incubated with the eNOS inhibitor, L-NAME. In addition, the mechanical denudating of endothelium significantly reduced the relaxation. These results indicate that stimulation of NO production, which relaxes smooth muscle by increasing cyclic GMP levels, and of eNOS is associated with the relaxation in RA rings.

The venodilating effect of chloroquine on human hand veins was mediated by the endothelium as shown by Abiose et al. Further inhibition of chloroquine-produced relaxation by coinfusion of H_1_/H_2_-receptor antagonist and NOS antagonist made the researchers suggest that chloroquine may partly act by stimulating histamine release [9]. Chloroquine was also shown by Ghigo et al. to stimulate NOS activity in murine, porcine, and human endothelial cells [19]. In one study, the effect of HCQ on vascular reactivity in systemic lupus erythematosus (SLE) mouse model was investigated. It was found that HCQ was able to restore vascular response to acetylcholine by increasing NO production, an effect that was abolished by L-NAME [13]. In a study examining the effects of chloroquine on the coronary artery of control and diabetic mice, the researchers found that chloroquine increased endothelium-dependent relaxation of the diabetic coronary arteries by enhancing EDHF pathway [20]. However, we found that indomethacin did not change the HCQ-induced relaxation of endothelium-intact RA rings, confirming that such relaxation may not be induced by prostaglandins or by concomitant production of prostacyclin.

Interestingly, only modest vasodilation effect of HCQ was observed in lower concentrations of HCQ solutions such as 10^−6^ M and 10^−5^ M, as relaxation of HCQ was little inhibited at 10^−5^ M and 10^−6^ M concentrations in the endothelium-denuded and L-NAME incubated RA rings.

We are suggesting that HCQ may not directly exert its effect on Ca^2+^channels to inhibit Ca^2+^ influx both in endothelial and smooth muscle cells. Rather, it is highly likely that HCQ may trigger an external pathway that indirectly signals the Ca^2+^ ion channels to stop Ca^2+^ influx and it would take greater concentrations of HCQ for trafficking the signal to the Ca^2+^ ion channels. Several investigators have highlighted the principal role of Ca^2+^ channels in bringing about vascular relaxation by chloroquine. Wu et al. concluded that chloroquine blocks store-operated, receptor-operated, and voltage-operated Ca^2+^ channels in pulmonary artery smooth muscle cells [21]. HCQ has been described by Capel et al. to inhibit multiple ion channels in sinoatrial node, including: funny current, rapid delayed rectifier potassium current, and L-type Ca^2+^ ion current leading to a significant reduction in spontaneous action potential firing rate [15]. In the present study, contractile responses to Ca^2+^ of RA rings exposed to KCl was used to identify agents which possess Ca^2+^ channel antagonist actions. The reason is that KCl depolarizes cell membranes and opens voltage-gated Ca^2+^ channels to permit extracellular Ca^2+^ to enter the cytosol and produce contractions [22]. To explore the involvement of Ca^2+^ channels in HCQ-produced vascular relaxation, we suspended RA rings in Ca^2+^-free Krebs solution, incubated HCQ in four different concentrations (10^−6^–10^−3^ M), then added CaCl_2_ (10^−5^–10^−2^) in a cumulative manner. We observed that the higher the incubated HCQ concentration is, the lower the contraction induced by CaCl_2_ is. In addition, after incubation of nilvadipine blocking L-type Ca^2+^ channels in vascular muscle cells [23], HCQ produced relaxation like the control group in RA rings precontracted with PE. This is in concordance with a previous study that demonstrated that chloroquine induces vasodilation in rat aorta at high concentration (>10^−4^ M) via inhibiting voltage-dependent L-type Ca^2+^ channel [24]. Our results suggested that HCQ can also produce vascular relaxation by blocking Ca^2+^ channels and preventing Ca^2+^ influx in both endothelium-denuded and -intact RA. The relaxation mediated by the Ca^2+^ channels was observed to be more evident at 10^−4^ M and 10^−3^ M HCQ concentrations that are unattainable at clinical blood concentrations [25].

Five types of K^+^ channels are present in vascular smooth muscles, and they play a crucial role in regulating vascular tone. Activation of K^+^ channels is a key mechanism of vasorelaxation caused by many drugs [26], such as sirolimus, levosimendan, pinacidil and propofol [26–29]. The activation of K^+^ channels lead to K^+^ efflux, membrane hyperpolarization and subsequent vascular relaxation [12]. BK_Ca_ plays an important role in regulating vascular tone, especially in conduit arteries. The relaxation induced by BK_Ca_ activation of some drugs in the vascular smooth muscles has been demonstrated such as propofol-internal mammary artery [26] and -rat aorta [14], and levosimendan-umbilical artery [30]. To illuminate the possible roles of K^+^ channels in relaxation of pulmonary artery produced by chloroquine, Wu et al. used blockers of BK_Ca_ and K_V_ channels, and stated that their effects were unnoticeable [21]. Whether or not HCQ activates K^+^ channels in RA rings has not been studied as far as we know. Accordingly, we incubated RA rings in Krebs solution containing blockers of BK_Ca_, K_ATP_, K_V_, and K_IR_ channels. RA rings were precontracted by the addition of PE, then HCQ (10^−6^–10^−3^ M) was added in a cumulative manner. HCQ produced vascular relaxation comparable to that obtained in the control RA rings. Thus, we could not find any significant association between K^+^ channels and vascular relaxation.

Pestana et al. reached a conclusion that inhibition of autophagy by chloroquine had restored NO levels on human umbilical vascular endothelial cells. They also found that incubating RA rings with chloroquine had resulted in endothelium-dependent relaxation in which the contribution of increased NO synthesis was confirmed [31]. Like chloroquine, HCQ is also known to be an inhibitor of autophagy by interference with lysosomal activity [3]. Transient receptor potential (TRP) channels are superfamily of cation channels; and, almost all functionally characterized transient receptor potential (TRP) channels are Ca^2+^ permeable, although not selective for Ca^2+^. TRPV4 (a member of the vanilloid subfamily of TRP channels) has been detected in both vascular smooth muscles and endothelial cells of rat cerebral artery and aorta [32]. Zhang et al. emphasized the importance of TRPV4 in mediating agonist-induced vascular dilation through endothelial-derived hyperpolarizing factors (EDHF) [33]. Whether inhibition of autophagy and TRPV4 activation by HCQ could contribute to the vascular relaxation was not examined in our study. Activation of TRPV4-EDHF and inhibition of autophagy are other possible relaxation pathways worthy to be investigated.

In summary, vascular smooth muscles can be relaxed by endothelium-dependent and endothelium-independent mechanisms. The present study shows that the relaxation of RA rings by HCQ is partly through NO and associated with blocking voltage gated Ca^2+^ channels. K^+^ channels have been shown to have no role in mediating relaxation. The observations from this study indicate that HCQ may be used in patients with noncardiac disease due to an ameliorative effect of HCQ on vascular diseases such as hypertension that causes vascular dysfunction.

## Footnotes

1
https://eda.nc3rs.org.uk/experimental-design-group
